# Stage-Specific Effects of Spatial Organization on Visual Search Under Clutter: Behavioral and Eye-Movement Evidence

**DOI:** 10.3390/jemr19050106

**Published:** 2026-09-21

**Authors:** Xinxin Wang, Jiaji Gao

**Affiliations:** School of Art and Design, Dalian Polytechnic University, Dalian 116034, China; 240621357000758@xy.dlpu.edu.cn

**Keywords:** visual search, eye tracking, visual clutter, spatial organization, target salience, gaze transitions, attention

## Abstract

Whether a well-organized scene facilitates visual search may depend on when in the search process the benefit is expressed. We tested this possibility in a virtual-office task in which 48 adults searched for targets varying in salience. Salience was manipulated between participants, whereas layout (grid, clustered, or random) and clutter (low, medium, or high) varied within participants. Mouse responses and eye position (120 Hz) were collected across 1296 target-present trials. Accuracy was broadly comparable across conditions, but reaction time (RT), time to first fixation (TTFF), fixation duration in the Target AOI, and fixation duration in the Clutter AOI each showed a three-way interaction. The transition data were particularly informative under high clutter. For grid layouts, clutter-to-target transitions occurred on 47.3% of transitions, clutter-to-clutter self-transitions on 20.5%, and the target was first reached after 0.89 intervening transitions on average. The respective values for random layouts were 11.2%, 64.2%, and 6.39. Thus, the benefit of the grid was not consistently present at initial target capture. It became most evident in the way gaze was redirected among regions as the search unfolded. Spatial regularity therefore seems to limit later search costs by making attentional reallocation and gaze-path correction more efficient.

## 1. Introduction

When target-relevant information is embedded among dense distractors, the search becomes slower and more effortful, a pattern which has been observed in real-world scenes and complex displays [1,2]. Yet a shorter search time does not reveal whether an arrangement helped observers first locate the target or complete the response after finding it. The unresolved question addressed here is whether spatial organization moderates clutter costs similarly across initial target acquisition, cumulative fixation allocation, and completed search. Comparing these outcomes within the same task makes that distinction testable.

### 1.1. Visual Search and the Rationale for the Manipulations

Search performance, however, is not determined by object count alone. Observers interpret a scene through its global organization, contextual expectations, semantic relations, and knowledge of the target [3,4,5,6,7,8,9,10,11,12,13,14,15,16,17]. Context can begin to guide the eyes before local details have been fully inspected [7,8]. Scene grammar and anchor objects further narrow likely target locations [10,11], while stimulus salience and top-down goals continue to compete for priority [9,16,17]. Recent evidence also implicates neural mechanisms in context-guided search [18]. Two scenes with the same number of objects may therefore differ in search difficulty because their spatial arrangements differ.

Several established measures describe visual clutter in terms of edge density, image complexity, or proto-object structure [19,20,21,22,23]. Yet these indices do not fully represent the arrangement of elements that may steer a search. Background clutter can affect search directly [24,25]. A previously encountered spatial context may also serve as a cue [26], and grouping changes when elements are connected or placed in regular arrangements [27,28]. Search guidance is also sensitive to instructions and to the weighting of target features [29]. For the present analysis, clutter and spatial organization are therefore treated as related but analytically distinct properties of the display.

Guided Search distinguishes the prioritization of locations from the processing needed to identify selected objects [17]. In the present design, target salience varied target-background distinctiveness, clutter varied local competition from non-target objects, and layout varied the regularity of relations among work units. A distinctive target may attract an early fixation without removing later response demands; spatial regularity may instead support orderly sampling of the scene. These mechanisms motivate comparing TTFF with RT and AOI fixation duration, rather than assuming that each manipulation has the same effect on every measure.

Eye movements make it possible to examine these influences as they unfold within a trial. AOI measures and fixation duration show where attention was allocated during interactive and scene-based tasks [30,31,32,33,34]. State and sequence models, in turn, describe the next step in the gaze path [35,36,37]. Together, these methods have been used to diagnose layout and interface problems in industrial, medical, consumer, and augmented-reality systems [38,39,40,41,42,43,44,45,46,47]. Related JEMR studies have applied eye-tracking measures to map search, time-limited tasks, and visual displays [48,49,50]. Of particular relevance here is the fact that a manipulation may leave initial target acquisition largely unchanged yet alter the later course of the search.

### 1.2. Hypotheses and Analysis Priorities

We tested these relationships in a controlled virtual office. The office served as the experimental context; the research question concerned how scene organization shapes search under clutter. Before data collection, we expected increasing clutter to lengthen RT and TTFF and increase fixation counts. We also expected the clutter-related RT increase to be smaller in grid than random layouts, with clustered layouts intermediate, and the layout advantage to be more pronounced for low-salience targets. The planned gaze-path account anticipated more Clutter-to-Clutter transitions in random scenes and more transitions towards the target in grid scenes under high clutter. These predictions did not specify a direction for every AOI-duration contrast.

The study was not preregistered. RT is the primary behavioral outcome, with target TTFF the key eye-movement outcome; accuracy and Target- and Clutter-AOI fixation durations provide secondary evidence. The outcome hierarchy is specified for the present report. Tests of the directional predictions above are distinguished from exploratory decompositions of individual conditions and interpretations of AOI-duration patterns. Interaction decompositions were exploratory; random-slope models assessed sensitivity to the random-effects specification.

## 2. Materials and Methods

### 2.1. Design and Participants

The experiment used a 2 (target salience: high, low; between participants) × 3 (spatial organization: grid, clustered, random; within participants) × 3 (visual clutter: low, medium, high; within participants) mixed design. Three viewing angles (left, center, and right) were used to increase stimulus diversity. The arrangement, clutter level, and target position were held constant across corresponding views; only the viewing direction changed. Viewing angle was treated as a control variable in trial-level models rather than as a focal experimental factor.

Forty-eight adults took part (24 men and 24 women; age range 25–45 years, M = 35.00, SD = 4.97). Thirty-six had a bachelor’s degree and 12 had a master’s degree. All reported general office experience, normal or corrected-to-normal vision, and no color-vision abnormality. Participants were stratified and randomly assigned to the high- and low-salience groups (*n* = 24 each). Group composition was kept approximately balanced for sex, age, and education. All participants provided written informed consent before the experiment. No participant was excluded from the study-level dataset. Participants were recruited through online advertisements and with assistance from university counselors and teachers. Each participant received CNY 100.

An a priori sample-size calculation was conducted using G*Power version 3.1.9.7 (Heinrich-Heine-Universität Düsseldorf, Düsseldorf, Germany) with the repeated-measures ANOVA within–between interaction option. The planning assumptions were f = 0.15, alpha = 0.05, power = 0.80, two groups, nine repeated conditions, a repeated-measures correlation of 0.50, and a nonsphericity correction of 0.75. The minimum total sample was 46; the final sample comprised 48 participants, 24 per group. This calculation approximated the overall interaction between salience group and the nine layout-by-clutter conditions, not the power of each specific interaction in the final mixed-effects models (Supplementary Methods S6) [51].

### 2.2. Virtual Office Stimuli

#### 2.2.1. Spatial Organization

The stimuli were constructed in a virtual office and were designed as a target-folder search task. Background architecture, basic furniture types, scene boundaries, target position, and task requirements were kept constant. Only the spatial relation among work units was changed. In the grid condition, workstations followed a stable row-and-column structure. In the clustered condition, workstations formed several compact local groups separated by clearer gaps. In the random condition, workstation locations and orientations did not form a stable global pattern. Figure 1 shows representative scenes from the three layout conditions. Additional stimulus matrices for the center and left viewing angles are provided in Figure S1.

#### 2.2.2. Visual Clutter and Target Salience

Visual clutter was manipulated by changing the number and local density of non-target objects in the task area. Low-, medium-, and high-clutter scenes contained 2, 7, and 15 added non-target objects, respectively. These values were chosen to create three separable levels within the capacity of the scene; they are not intended as general thresholds for office clutter. The target, scene category, and task remained unchanged across clutter levels.

The clutter manipulation was checked using a fixed image region of interest extracted from each base stimulus. Edge density was the primary image-level metric [19], supplemented by feature congestion and luminance standard deviation. Twenty-seven base images were included in this manipulation check (3 layouts × 3 clutter levels × 3 viewing angles). Edge density and feature congestion increased across clutter levels, whereas layout did not produce a significant main effect or interaction with clutter. Luminance standard deviation did not differ reliably across the experimental factors. Figure 2 shows the image-level procedure and Table 1 reports the inferential results.

Target salience was manipulated using an upright folder. The high-salience target was red and the low-salience target was black. The office scenes were dominated by gray, white, and dark surfaces. The red target therefore differed more strongly from its surroundings, whereas the black target was more similar to monitors and other dark regions. This manipulation was intended to create relative high- and low-salience conditions rather than to test a general advantage of red over other colors. Target-feature weighting and target-background similarity are known to influence visual search [16,17,25,28,29,52,53].

### 2.3. Eye-Tracking Apparatus and Procedure

Eye movements were recorded with a Tobii Pro X3-120 eye tracker (Tobii Pro AB, Danderyd, Sweden) at 120 Hz using Tobii Pro Lab software version 24.21.435.0 (Tobii Pro AB, Danderyd, Sweden). Stimuli were shown on a 23.8-inch display (1920 × 1080 pixels) at approximately 65 cm. Nine-point calibration was followed by validation; the acceptance criterion was a mean angular error no greater than 0.5 degrees, with errors inspected across validation locations. Calibration and validation were repeated after adjustment when this criterion was not met. Fixations were classified with the Tobii I-VT (Fixation) filter: velocity threshold 30 degrees/s, velocity window 20 ms, and a three-sample moving median. Average eye selection was used, taking binocular position when both eyes were available and the available eye otherwise. Missing samples were not interpolated. Adjacent fixations were merged with maximum separation of 75 ms and 0.5 degrees; fixations shorter than 60 ms were discarded. Invalid samples were not assigned to an AOI. Fixation-level records were exported for analysis (Supplementary Methods S6).

Target-present trials required at least 80% valid gaze samples over the scene-presentation interval. Twelve presentations failed this requirement and were each repeated once successfully. The failed records were replaced by their corresponding repeats, yielding 1296 retained records from 1308 target-present presentations, including repeats. This accounting excludes the target-absent trials.

Participants first completed consent and color-vision screening, followed by setup and calibration. They then completed three viewing-angle modules. Module order was counterbalanced across participants. Each module contained nine target-present trials, covering all layout × clutter combinations, and two blank trials. The full experiment therefore contained 33 trials: 27 target-present trials and 6 target-absent trials. Blank trials reduced the expectation that a target would always appear and were not included in the formal analyses.

Each trial began with a 1 s fixation point and drift correction. The office scene then appeared. On target-present trials, participants clicked the target folder with the mouse. On blank trials, they pressed the space bar. The maximum scene duration was 10 s. A target-present trial with no response within 10 s was coded as a timeout; a click on a non-target object was coded as an error.

The pre-task questionnaire recorded age, occupation, vision, and color-vision difficulties for sample description; these responses were not entered as predictors. After all search trials, participants retrospectively rated the workload associated with each image on six NASA-TLX dimensions using a 1–20 response scale. These ratings were not included in the present analyses. The inventory of collected measures and their reporting status is provided in Supplementary Methods S6. The eye-tracking setup and experimental procedure are illustrated in Figure 3.

### 2.4. AOIs and Outcome Measures

Eye-movement analysis used three mutually exclusive areas of interest (AOIs): Target, Clutter, and Background. The Target AOI covered the task-relevant folder. Multiple non-target object regions were combined into the Clutter state for analysis. All remaining scene regions were coded as Background. This coarse partition was chosen to separate target processing, sustained processing of irrelevant information, and general scene scanning while limiting the number of states in the transition model [30,31,32,33,34,35]. Target and Clutter boundaries followed the visible outlines of the target and designated non-target objects. Each fixation was assigned by its representative position within these mutually exclusive regions. Remaining valid positions inside the image were assigned to Background; invalid and off-screen positions were left unassigned.

Behavioral outcomes were accuracy and reaction time (RT). RT was the interval between scene onset and completion of the mouse-click response and was analyzed only for correct, valid target-present trials. Accuracy was coded 1 for a correct target click and 0 for an incorrect click or timeout. The eye-movement outcomes were TTFF on the Target AOI, total fixation duration in the Target AOI, and total fixation duration in the Clutter AOI. TTFF was used as an index of initial target acquisition. The two duration measures quantified cumulative fixation allocation over the scene-presentation interval; neither isolates a purely post-acquisition stage.

For the search-path analysis, successive fixation states were coded as Target (T), Clutter (C), or Background (B). Self-transitions within the same AOI class were retained, and sequences were never connected across trials. We analyzed the C-to-T transition probability, the C-to-C self-transition probability, and the number of transitions made before the target was first reached. These measures capture whether attention leaves irrelevant information and returns to the task-relevant region, rather than only whether the target is fixated at some point.

### 2.5. Statistical Analysis

Trial-level records were retained for the main inferential analyses. Accuracy used all 1296 retained target-present trials; correct clicks were coded 1 and errors or timeouts 0. Continuous outcomes were natural-log transformed and analyzed for correct trials with finite, positive values. RT, TTFF, and Clutter-AOI fixation duration each included 1068 trials. Target-AOI fixation duration included 1064 after exclusion of three negative values and one zero. Nonpositive durations were excluded because their logarithms are undefined. All primary models included 48 participants.

Equation (1) specifies the model for log-transformed continuous outcomes, estimated by restricted maximum likelihood. Equation (2) specifies the binomial model for response accuracy, estimated by maximum likelihood with a logit link.

(1) $$ln(Y_{ij})={x_{ij}}^{T}\beta +b_{i}+\varepsilon_{ij}$$

(2) $$logit[P(A_{ij}=1\;|\;b_{i})]={x_{ij}}^{T}\beta +b_{i}$$

Here, *i* indexes participants and *j* trials; *A* is response accuracy, and *x* contains the intercept, salience, layout, clutter, all interactions among these three factors, and viewing angle. The participant intercept *b_i_* and continuous residual *ε_ij_* are Gaussian. Sum-to-zero coding was used for all factors; level orders and full contrast definitions are given in Supplementary Methods S6. Omnibus fixed effects were tested with Wald chi-square tests. A participant random intercept provided a parsimonious primary specification with only three observations per layout-by-clutter cell; participant-specific layout and clutter slopes were assessed in sensitivity analyses. Simple comparisons used Bonferroni adjustment, with alpha = 0.05.

The primary models were fitted in Python 3.12.14 with NumPy 2.3.5 and SciPy 1.17.0. Accuracy used 25-point Gauss–Hermite quadrature; fixed-effect uncertainty for the primary models was obtained from the observed joint Hessian. Supplementary Methods S6 detail the model specifications and test definitions. Interaction decompositions compare high-to-low clutter ratios between layouts and then between salience groups. Estimates and pointwise 95% CIs are reported in Table S7; adjusted *p* values refer to the stated contrast families.

Gaze transitions were evaluated with generalized estimating equations (GEEs) that accounted for repeated observations within participants. Binomial models were used for C-to-T and C-to-C probabilities. Counts of transitions preceding the first Target-AOI visit were converted with ln(1 + x) and fitted with a Gaussian GEE. Predictors in all three models were target salience, spatial organization, visual clutter, and their interactions. The analysis follows state-based approaches for describing scanpaths and task-sensitive gaze sequences [35,36,37]. An exchangeable working correlation and participant-clustered robust covariance were used. Sample sizes, estimating equations, and interaction contrasts are detailed in Supplementary Methods S6.4 and Table S9.

A sensitivity analysis tested whether sparse correct-trial counts within some participant × layout × clutter cells affected the continuous-outcome results. We first retained cells containing at least two valid correct trials, averaged the log-transformed outcome within each cell, and refitted the 2 × 3 × 3 mixed-effects model with participant as a random intercept. A stricter analysis retained only cells in which all three viewing-angle trials were valid. Viewing angle was not entered in these cell-level sensitivity models because the three views had already been combined.

Random-slope sensitivity models retained the same fixed effects and trial sets, adding two categorical layout slopes and two clutter slopes per participant, with either independent or correlated random coefficients. Continuous models used REML; the accuracy sensitivity model used independent slopes and a Laplace approximation. Convergence and covariance boundary diagnostics are reported in Table S8.

## 3. Results

### 3.1. Manipulation Checks and Trial Outcomes

The target-salience manipulation produced the intended difference in early target acquisition. Participant-level mean TTFF was 293.81 ± 16.39 ms in the high-salience group and 430.56 ± 21.17 ms in the low-salience group. The Welch test was significant, t(43.28) = −25.02, *p* < 0.001. Accuracy was similar in the two groups (82.56% and 82.25%). In the formal accuracy model, the main effect of target salience was not significant, with a Wald chi-square (1) = 0.070, *p* = 0.791. The color manipulation therefore altered early visual acquisition without producing a general change in final accuracy.

Across the 1296 target-present trials, 1068 responses were correct (82.4%) and 228 were errors (17.6%). Every error was a click on a non-target object within the 10 s response window. No timeout occurred. The accuracy model therefore retained all target-present trials. None of the experimental main effects or interactions reached significance for accuracy (all *p* > 0.05). Viewing angle had a small but significant control effect; Wald chi-square (2) = 7.137, *p* = 0.028. Thus, the manipulations altered how quickly participants searched and how their eyes moved, but not whether they ultimately selected the correct object. Complete condition-level descriptive statistics for accuracy, RT, TTFF, Target-AOI fixation duration, and Clutter-AOI fixation duration are provided in Table S1.

The 1296 retained records follow replacement of the 12 quality-failed presentations described in Section 2.3. After the 228 incorrect responses were excluded, the continuous-model sample was 1068, except for Target-AOI duration (1064). Table S3D separates acquisition quality screening from response and outcome-specific exclusions. The full Wald chi-square statistics and associated *p* values are summarized in Table 2.

### 3.2. Reaction Time

RT showed a significant target salience × layout × clutter interaction, Wald chi-square (4) = 129.347, *p* < 0.001. Search time increased as clutter rose, but the size of that increase depended strongly on layout and salience. Grid scenes were generally completed more quickly than random scenes. The largest mean RT occurred in the low-salience, random, high-clutter condition (4804.70 ms). Within both salience groups, RT increased significantly across clutter levels for all three layouts; Bonferroni-corrected pairwise comparisons among clutter levels were significant (all *p* < 0.001). The increase was steepest in the random layout.

The high-to-low clutter ratio in the grid relative to random layouts was 1.047 (95% CI [0.971, 1.129]) under high salience and 0.664 [0.617, 0.715] under low salience. Comparing these ratios between low and high salience gave 0.634 [0.571, 0.705] (Bonferroni-adjusted *p* < 0.001). These contrasts describe a specific component of the interaction; Table S7 provides all layout-pair estimates. Reaction times increased with visual clutter and differed across spatial organization conditions (Figure 4).

### 3.3. Eye-Movement Time Measures

#### 3.3.1. Time to First Fixation

TTFF also showed a significant three-way interaction, Wald chi-square (4) = 64.023, *p* < 0.001. The pattern differed from RT. In the high-salience group, TTFF increased with clutter in the grid and clustered layouts (all pairwise *p* < 0.001). The random layout did not follow this monotonic pattern: TTFF was highest at medium clutter and lowest at high clutter, with all three levels differing after correction (all *p* < 0.001). In the low-salience random layout, low clutter produced a shorter TTFF than medium or high clutter (both *p* < 0.001), whereas medium and high clutter did not differ (*p* = 1.000). Thus, the layout that produced the shortest early-acquisition time under high clutter was not necessarily the layout that produced the fastest completed response.

The high-to-low clutter ratio in grid relative to random layouts was 3.618 (95% CI [3.212, 4.075]) under high salience and 1.986 [1.766, 2.235] under low salience. Comparing these ratios between low and high salience gave 0.549 [0.464, 0.649] (Bonferroni-adjusted *p* < 0.001). These contrasts describe a specific component of the interaction; Table S7 provides all layout-pair estimates. Time to first fixation on the target varied across spatial organization and visual-clutter conditions (Figure 5).

#### 3.3.2. Fixation Duration in the Clutter AOI

Clutter-AOI fixation duration showed a significant three-way interaction, Wald chi-square (4) = 29.401, *p* < 0.001. Dwell time in irrelevant regions generally increased with clutter in the clustered and random layouts. The clearest cost occurred in the low-salience random condition, where mean Clutter-AOI fixation duration reached 3842.49 ms under high clutter and was significantly longer than under low or medium clutter (both *p* < 0.001). The grid condition did not show the same sustained increase. Several adjacent clutter levels were not significantly different within that layout. This measure therefore separated structured from random scenes more consistently than TTFF did.

The high-to-low clutter ratio in grid relative to random layouts was 0.357 (95% CI [0.308, 0.414]) under high salience and 0.237 [0.204, 0.274] under low salience. Comparing these ratios between low and high salience gave 0.663 [0.538, 0.817] (Bonferroni-adjusted *p* < 0.001). These contrasts describe a specific component of the interaction; Table S7 provides all layout-pair estimates. Total fixation duration in the Clutter AOI differed across spatial organization and visual-clutter conditions (Figure 6).

#### 3.3.3. Fixation Duration in the Target AOI

Target-AOI fixation duration also showed a three-way interaction, Wald chi-square (4) = 31.297, *p* < 0.001. In the high-salience group, fixation time on the target generally increased with clutter for grid and clustered layouts, whereas the random layout showed lower target fixation time at high than at medium clutter. In the low-salience random condition, low clutter produced shorter Target-AOI fixation duration than medium and high clutter (adjusted *p* = 0.014 and *p* = 0.002, respectively); medium and high clutter did not differ (*p* = 1.000). Target-AOI fixation duration was not treated as an isolated index of efficiency, because a longer fixation may indicate either difficulty or additional verification. Its pattern was therefore evaluated alongside RT, TTFF, and time spent in the Clutter AOI. Complete Bonferroni-adjusted clutter-level pairwise comparisons for RT and the eye-movement time measures are reported in Table S2.

The high-to-low clutter ratio in grid relative to random layouts was 3.632 (95% CI [2.934, 4.497]) under high salience and 2.042 [1.654, 2.522] under low salience. Comparing these ratios between low and high salience gave 0.562 [0.416, 0.759] (Bonferroni-adjusted *p* < 0.001). These contrasts describe a specific component of the interaction; Table S7 provides all layout-pair estimates.

### 3.4. AOI Distribution and Representative Scanpaths

Figure 7 provides a descriptive view of the same pattern observed in the trial-level models. With clutter held high, gaze in grid scenes was largely confined to the target and adjacent work units. In clustered scenes, fixations gravitated toward the central grouping. Random scenes, by contrast, dispersed fixations across more work units and produced visibly longer scanpaths. The heatmaps show what the modeled differences looked like in representative trials; statistical inference rests on the trial-level analyses.

### 3.5. Sensitivity Analysis

The trial-level pattern was not driven by a small number of sparse cells. Of 432 participant × layout × clutter cells, 237 contained three correct trials, 164 contained two, 29 contained one, and two contained none. After cells with fewer than two valid correct trials were removed, the three-way interactions remained significant for RT, TTFF, Target-AOI fixation duration, and Clutter-AOI fixation duration: Wald chi-square (4) = 115.420, 69.130, 27.858, and 25.307, respectively (all *p* < 0.001). The stricter analysis using only complete three-trial cells gave the same conclusion, with corresponding values of 86.337, 43.847, 27.253, and 20.413 (all *p* < 0.001). The valid-trial distribution, core sensitivity results, and model-sample audit are provided in Table S3.

The layout-by-clutter and three-way interactions also remained significant in the independent- and correlated-random-slope models. For correlated slopes, the respective Wald chi-square (4) values were 119.317 and 131.741 for RT, 775.452 and 64.104 for TTFF, 233.949 and 31.802 for Target duration, and 866.358 and 30.585 for Clutter duration (all *p* < 0.001). Optimizers converged, although every continuous random-slope model had boundary variance components or a rank-deficient covariance matrix. The accuracy three-way interaction remained nonsignificant with independent slopes, chi-square (4) = 6.957, *p* = 0.138. Table S8 reports the diagnostics and covariance convention used for these comparisons.

### 3.6. Gaze-State Transitions

The state-transition models provided a different view of the layout effect. C-to-T probability was affected by layout, Wald chi-square (2) = 64.719, *p* < 0.001, and clutter, Wald chi-square (2) = 8.140, *p* = 0.017. Their interaction was also significant; Wald chi-square (4) = 138.595, *p* < 0.001. C-to-C self-transition probability showed the same broad pattern: layout, Wald chi-square (2) = 92.251, *p* < 0.001; clutter, Wald chi-square (2) = 6.655, *p* = 0.036; layout × clutter, Wald chi-square (4) = 197.320, *p* < 0.001. Neither probability measure showed a significant three-way interaction with salience.

The number of transitions before the target was initially reached was influenced by layout, Wald chi-square (2) = 87.558, *p* < 0.001, clutter, Wald chi-square (2) = 21.167, *p* < 0.001, and the layout × clutter interaction, Wald chi-square (4) = 212.272, *p* < 0.001. The salience × layout interaction was also significant; Wald chi-square (2) = 8.617, *p* = 0.013. The three-way interaction was not significant; *p* = 0.170. Condition-level gaze-transition summaries are provided in Table S4.

The high-clutter condition makes the pattern especially clear. The C-to-T transition probability was 47.3% in grid scenes, 28.2% in clustered scenes, and 11.2% in random scenes. The C-to-C self-transition probability was 20.5%, 36.5%, and 64.2%, respectively. Before first reaching the target, participants made an average of 0.89, 2.20, and 6.39 transitions in the same three layouts. All pairwise layout differences for these high-clutter summaries were significant after Bonferroni correction (*p* ≤ 0.007). Figure 8 shows the transition matrices and corresponding transition networks. Complete transition matrices across spatial-organization and visual-clutter conditions are provided in Table S5. The GEE results for gaze-path measures are summarized in Table 3.

The Grid/Random ratio of high-to-low clutter odds ratios was 7.665 (95% CI [4.929, 11.922]) for C-to-T and 0.150 [0.097, 0.233] for C-to-C transitions. The corresponding ratio for the geometric mean of one plus the pre-target transition count was 0.286 [0.226, 0.362]. These contrasts average equally over salience groups on the model scale (all adjusted *p* < 0.001). Table S9 also reports the salience-dependent contrasts and their uncertainty.

## 4. Discussion

### 4.1. Visual Clutter and Spatial Organization

The search became slower as visual clutter increased, but the cost was not uniform across layouts. The random configuration deteriorated most sharply under high clutter. Accuracy, by contrast, remained fairly stable. This separation is consistent with work showing that clutter often changes search time and attentional allocation before it produces a large loss in final response accuracy [1,2,54,55]. In the present task, spatial organization changed how much processing was required to arrive at a response, not simply whether the target could eventually be found.

Scene structure offers a plausible explanation. Context, semantics, and global organization can constrain the region that is searched and influence the next fixation [7,8,9,10,11,12,13,14,15,18,52,53,56,57,58,59,60,61,62,63,64]. A regular layout supplies repeated positional relations. Those relations may help gaze return to task-relevant space after an irrelevant object has been sampled. The result should not be read as a claim that grids are universally superior. What the data support is narrower: under dense visual competition, predictability reduced the additional search cost associated with random spatial arrangement.

These findings qualify the link between contextual guidance and search efficiency [7,8,17]. The arrangement that minimized completed response time did not necessarily minimize first-target-fixation latency. Clutter measures describe competition in the image [1,19], whereas the present outcome comparison shows that the consequences of that competition also depend on how performance is measured. The contribution is therefore the divergence among acquisition, fixation allocation, and completion within a common set of scenes.

### 4.2. Early Acquisition and Later Search Organization

TTFF and RT did not tell the same story. Under high clutter, a salient target in the random layout could be fixated relatively early, yet the response remained slow and clutter dwell remained high. Early acquisition was therefore not equivalent to efficient completion. Similar distinctions are implicit in scene-viewing work showing that fixation duration, contextual guidance, and subsequent eye movements continue to reflect task demands after an object has entered the foveal or near-foveal region [13,14,15,32,34,52,53,57,58,59,60,61,62,63,64].

The transition analysis further describes how search paths differed. In high clutter, gaze moved from Clutter to Target on 47.3% of transitions in the grid condition but only 11.2% in the random condition. Repeated Clutter-to-Clutter transitions showed the reverse pattern, 20.5% versus 64.2%. The number of transitions before the first target visit also rose from 0.89 to 6.39. These differences cannot be reduced to target color because the target was held constant across layouts. They are more compatible with a structural account in which contextual cueing, grouping, and background organization affect how gaze is redirected [25,26,27,28,29]. Because these transitions span search and the target-reach count precedes the first target visit, they do not directly isolate a later processing stage. Attentional reallocation remains a mechanism consistent with the pattern, rather than a separately measured post-acquisition interval.

### 4.3. Target Salience and Boundary Conditions

Target salience changed the speed of initial acquisition, as intended. It also entered the three-way interactions for the continuous outcomes. The manipulation, however, used only two folder colors. It should be interpreted as a contrast between relatively stronger and weaker target-background distinctiveness, not as a general claim about red and black. Search guidance is known to vary with target features, instructions, background similarity, and scene constraints [16,17,25,28,29,52,53].

The gaze-transition outcomes were less dependent on salience. Their three-way interactions were not significant, although the magnitude of layout differences varied descriptively between salience groups. This is useful because it separates two design levers that are sometimes treated as interchangeable. Increasing target salience may assist early orientation. A more predictable layout can still matter later, when attention has to disengage from irrelevant information and resume a task-directed path.

### 4.4. Implications for Applied Eye-Movement Research

A single eye-movement metric would have missed part of the effect. TTFF captured the first target visit, whereas clutter dwell and transition probabilities described what happened around that event. AOI and fixation measures have long been used in human–computer interaction and information visualization [30,31,32,33,34,36,37]. Applied design studies extend the same logic to equipment displays, control interfaces, mobile applications, medical software, and augmented-reality instructions [38,39,40,41,42,43,44,45,46,47]. Recent JEMR work on cartographic search, successive search under time pressure, and terminal displays also illustrates the breadth of this process-oriented approach [48,49,50].

For office work, the practical implication should remain modest. Stable rows or clearly bounded groups may reduce repeated scanning when task-relevant items are embedded in a dense field. This does not mean that every workspace should be converted to a grid. Office-performance research shows that spatial arrangements involve competing demands and user needs [65,66,67,68,69,70,71,72,73,74,75,76,77]. The present experiment supports the possibility that spatial predictability can reduce the attentional cost of repeated target search when clutter cannot be fully removed.

### 4.5. Limitations and Future Work

The task used static virtual-office scenes and a screen-based response. Real search also involves head and body movement, occlusion, reaching, object handling, and memory for changing locations. Active-viewing work shows that gaze behavior can differ when observers move through an environment rather than inspect a fixed image [78]. Replication in immersive or physical workspaces is therefore needed before the effect is generalized to everyday office behavior.

Each participant contributed three target-present repetitions to a given layout × clutter cell across viewing angles. The sensitivity analyses reduced concern that sparse cells created the main interactions, but additional repetitions would improve participant-level stability. The AOI model was intentionally coarse as well. Target, Clutter, and Background capture the broad task structure, yet they merge many distinct objects. More detailed AOIs, saccade direction, path length, and time-resolved sequence models could identify which local scene relations produce the largest interference cost [31,34,35,36,37].

A final limitation concerns target salience. The manipulation was validated within the main experiment rather than in an independent pretest, and only color was varied. Future studies should manipulate contrast, shape, size, and target-background similarity separately. Repeated exposure also deserves attention. Contextual cueing can become stronger as a scene is learned [26], which may alter the stage at which spatial organization begins to influence gaze.

## 5. Conclusions

Spatial organization changed how visual clutter affected search in a virtual office. Accuracy remained broadly stable, but RT and eye-movement measures did not. Random layouts became especially costly as clutter increased. A regular grid reduced sustained fixation on irrelevant regions and produced more efficient gaze transitions under high clutter.

Across measures, the layout effect depended on the stage of the search being considered. Regular spatial organization did not consistently hasten the first acquisition of the target. The advantage became more apparent as gaze disengaged from clutter, shifted back toward relevant locations, and corrected its path through the scene. TTFF alone therefore captured only part of the efficiency difference, a point that matters when eye-movement studies use first-fixation latency as their primary outcome. In practical terms, predictable arrangements may still ease search where reducing the total amount of visual information is not feasible.

## Figures and Tables

**Figure 1:**
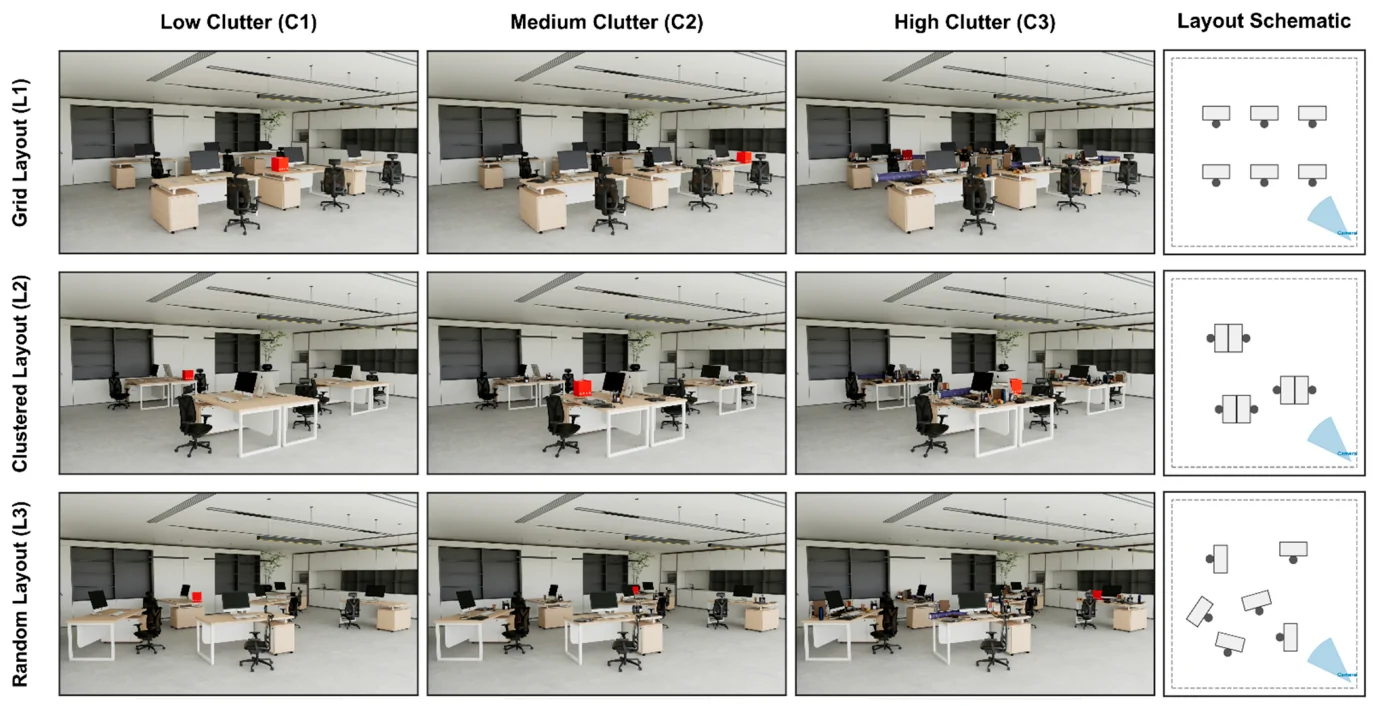
Representative stimulus matrix for the grid, clustered, and random spatial organizations across low, medium, and high visual clutter. The schematic column illustrates the spatial relation among work units. The displayed examples use the high-salience target; the low-salience condition differed only in target color.

**Figure 2:**
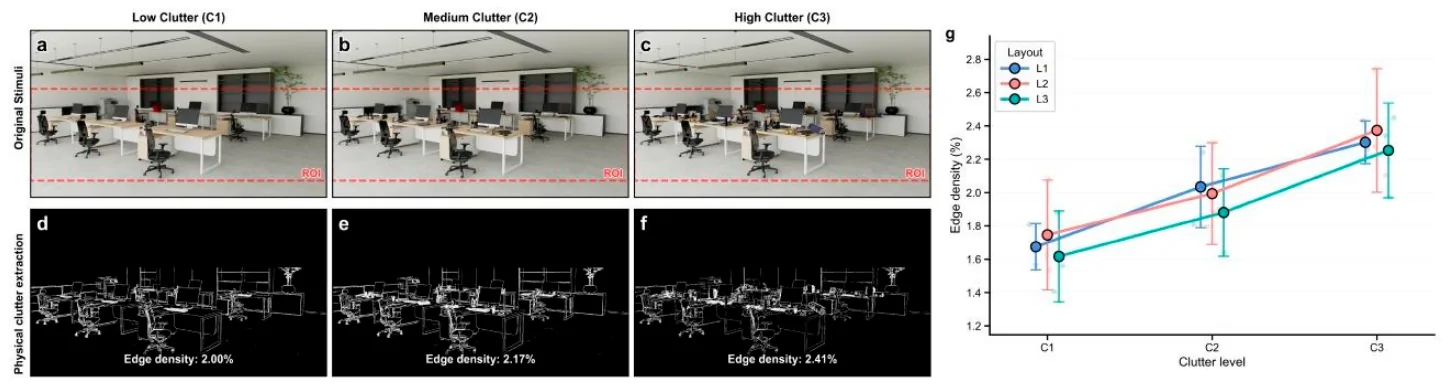
Image-based validation of the visual-clutter manipulation. (**a**–**c**) Representative low-, medium-, and high-clutter scenes (C1–C3), respectively, with red dashed lines indicating the boundaries of the fixed task region of interest (ROI). (**d**–**f**) Corresponding edge-extraction maps for the low-, medium-, and high-clutter scenes, respectively. (**g**) Edge density across layouts and clutter levels. The red dashed lines indicate the boundaries of the fixed task region of interest (ROI).

**Figure 3:**
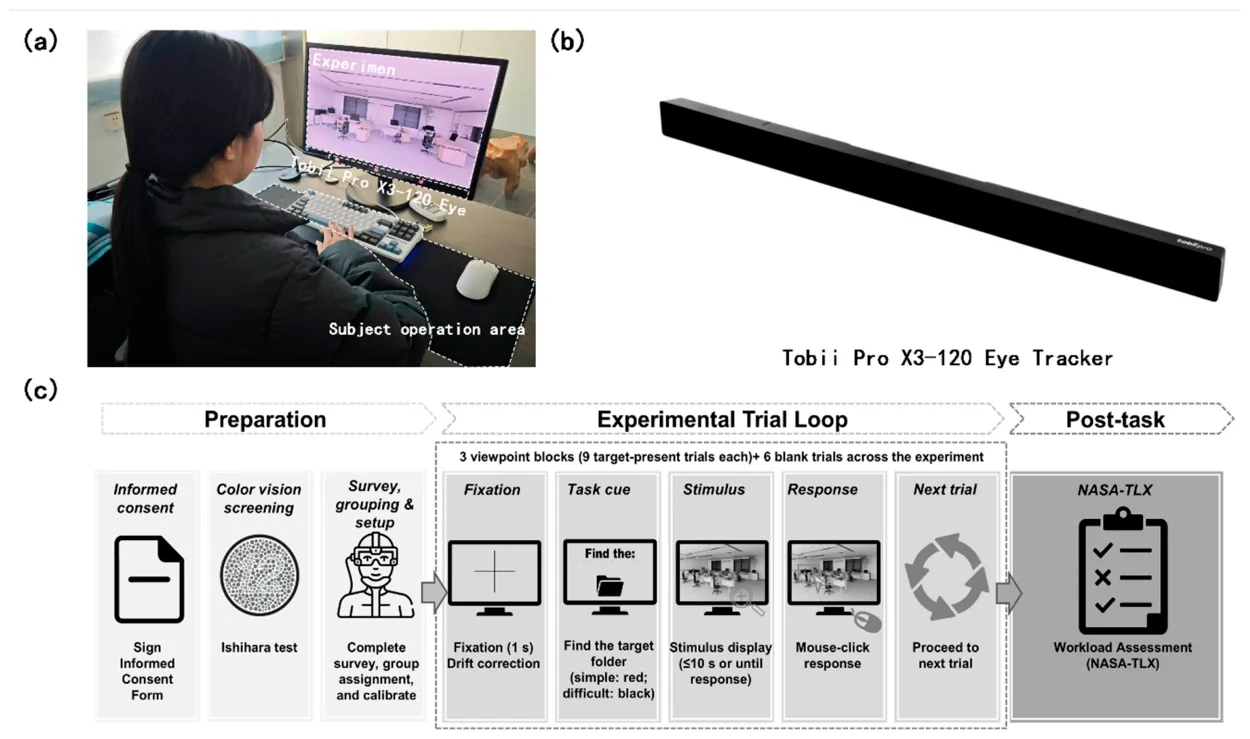
Eye-tracking setup and task procedure. (**a**) Experimental setting; (**b**) Tobii Pro X3-120 eye tracker; (**c**) preparation and repeated trial sequence. The pre-task survey collected sample characteristics; NASA-TLX ratings were completed retrospectively for each image after all search trials.

**Figure 4:**
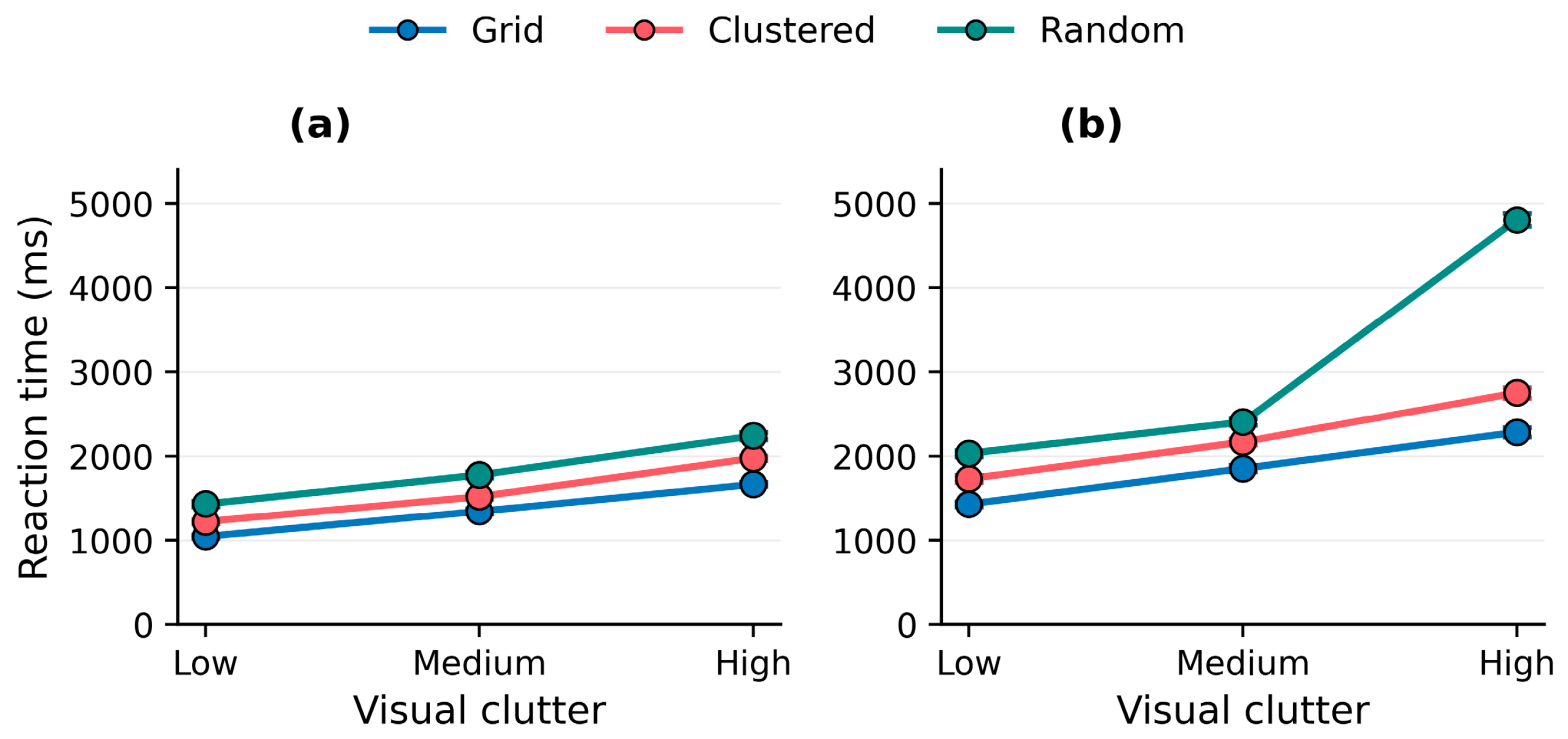
Reaction time across spatial organization and visual-clutter conditions (mean ± standard error). (**a**) High target salience; (**b**) low target salience.

**Figure 5:**
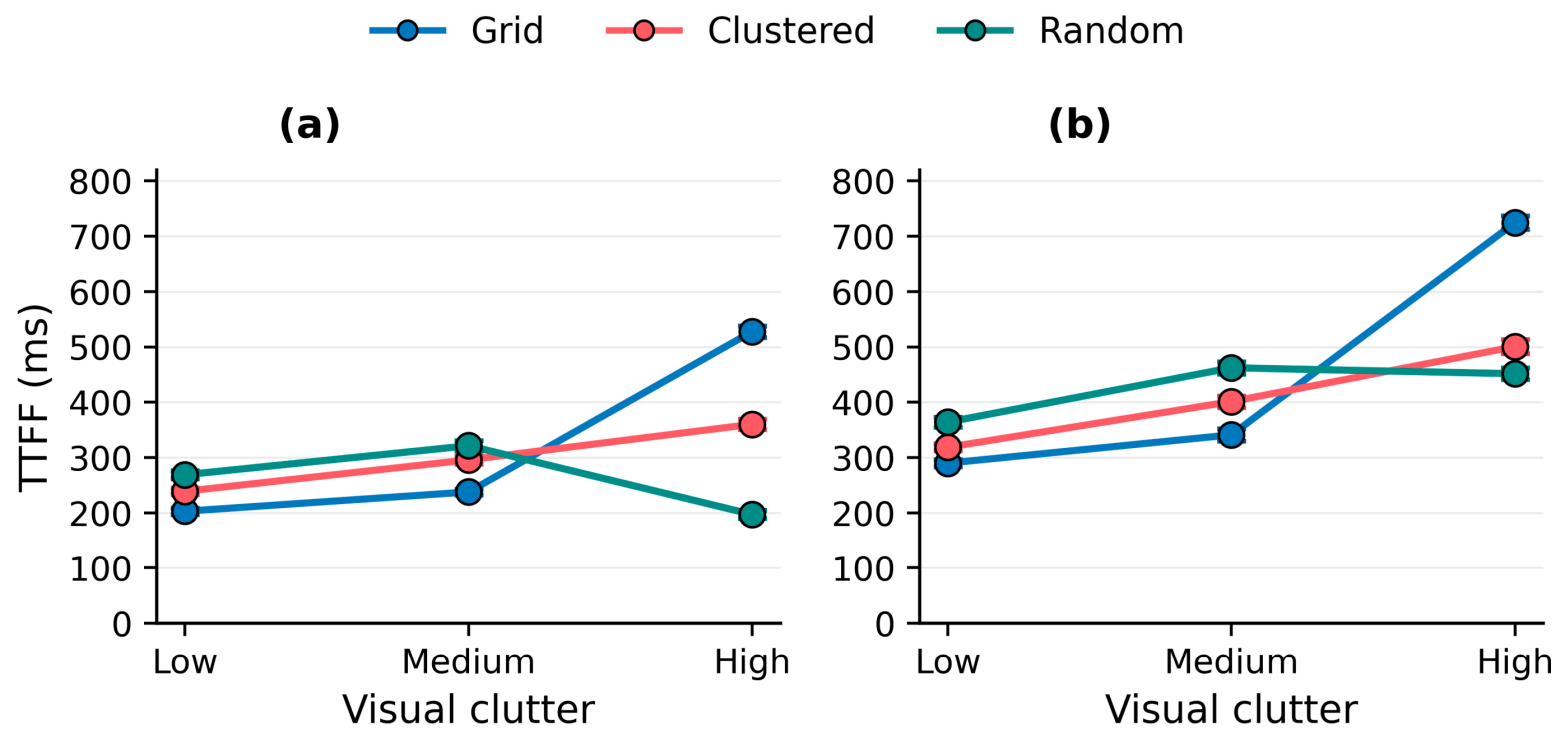
Time to first fixation on the target across spatial organization and visual-clutter conditions (mean ± standard error). (**a**) High target salience; (**b**) low target salience.

**Figure 6:**
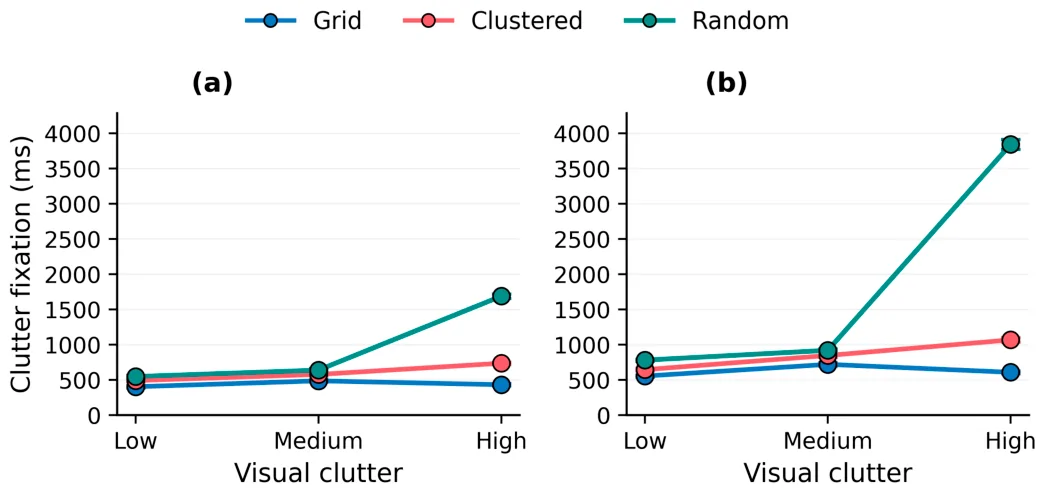
Total fixation duration in the Clutter AOI across spatial organization and visual-clutter conditions (mean ± standard error). (**a**) High target salience; (**b**) low target salience.

**Figure 7:**
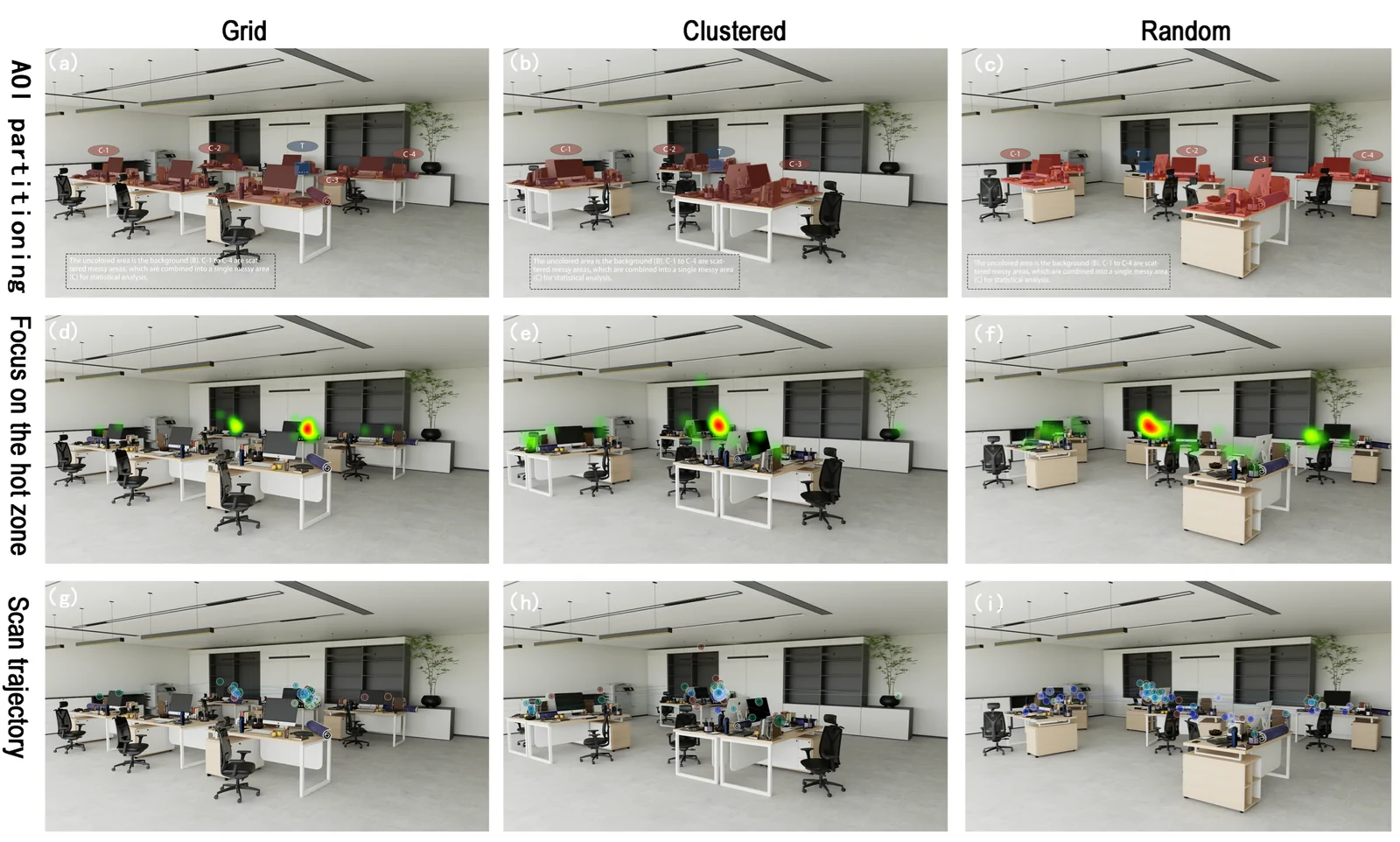
AOI maps, fixation heatmaps, and representative scanpaths under high visual clutter for grid, clustered, and random layouts. (**a**–**c**) AOI maps showing the spatial partitioning of target and clutter regions in grid, clustered, and random layouts, respectively. (**d**–**f**) Fixation heatmaps showing gaze distributions under the three layouts, respectively. (**g**–**i**) Representative scanpaths illustrating gaze transitions under grid, clustered, and random layouts, respectively. Colored markers indicate the temporal sequence of fixations. The panels show representative spatial patterns in gaze allocation; statistical conclusions were drawn from the inferential models.

**Figure 8:**
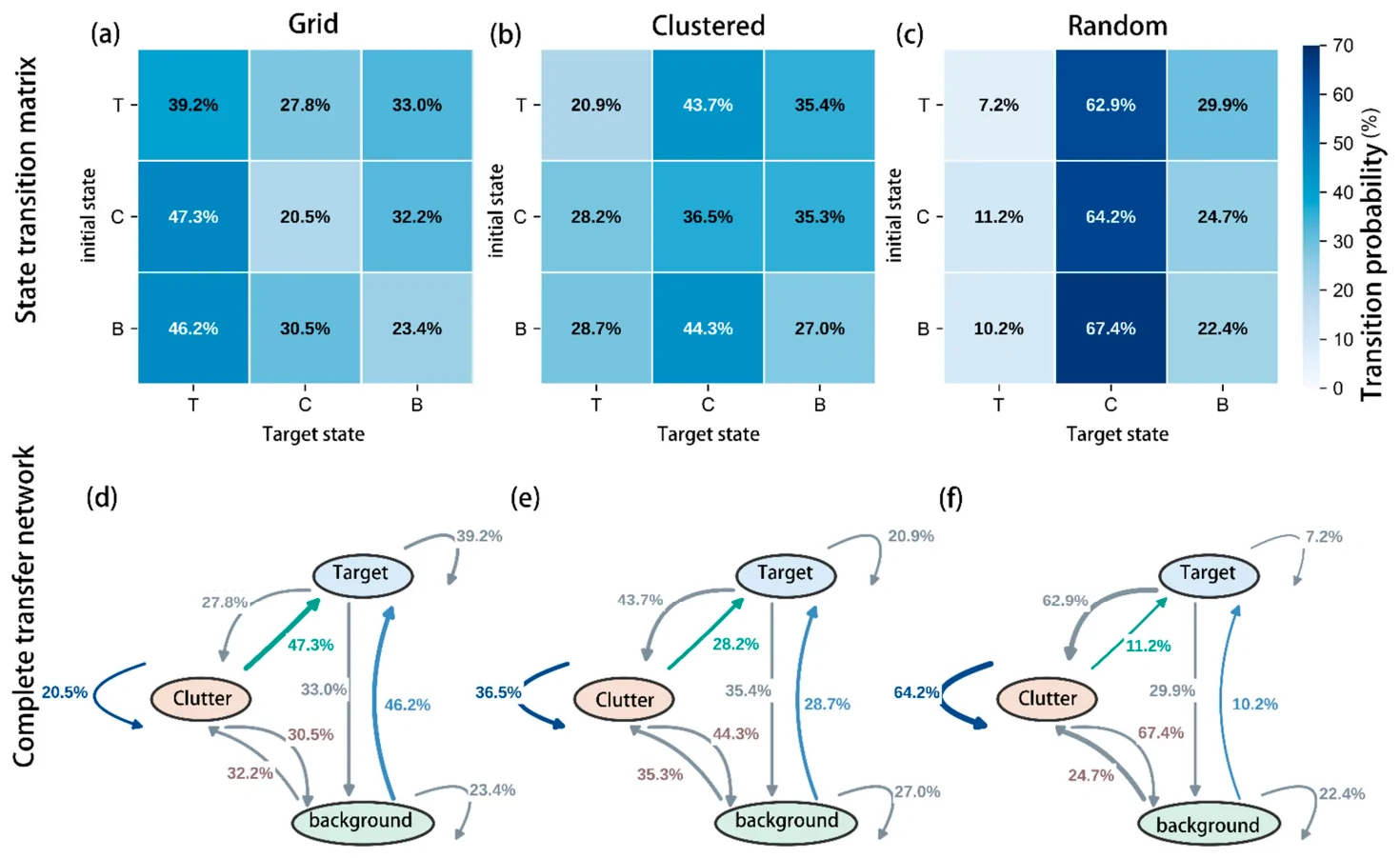
Search paths under high visual clutter. (**a**–**c**) State-transition matrices for grid, clustered, and random layouts; (**d**–**f**) the corresponding transition networks among Target (T), Clutter (C), and Background (B). The highlighted pattern shows that grid scenes more often redirected gaze from clutter toward the target, whereas random scenes produced more repeated processing within clutter. In the transfer networks (**d**–**f**), green arrows indicate clutter-to-target transitions, blue arrows indicate target-related return transitions, and gray arrows indicate other state transitions.

**Table 1:** Image-level manipulation check for visual clutter.

Metric	Effect	*df*	*F*	*p*	Partial Eta-Squared
Edge density	Layout	2.18	0.16	0.855	0.017
	Clutter	2.18	11.28	<0.001	0.556
	Layout × clutter	4.18	0.00	0.999	0.001
Feature congestion	Layout	2.18	2.44	0.115	0.213
	Clutter	2.18	13.90	<0.001	0.607
	Layout × clutter	4.18	0.26	0.897	0.055
Luminance SD	Layout	2.18	0.09	0.918	0.009
	Clutter	2.18	1.72	0.207	0.161
	Layout × clutter	4.18	0.01	0.999	0.002

**Table 2:** Wald chi-square tests for target salience (S), spatial layout (L), visual clutter (C), and their interactions. Each cell reports chi-square; *p*. Continuous outcomes were log transformed before fitting linear mixed-effects models.

Outcome	Salience	Layout	Clutter	S × L	S × C	L × C	S × L × C
Accuracy	0.070; 0.791	3.673; 0.159	0.092; 0.955	0.094; 0.954	0.403; 0.817	2.291; 0.682	6.904; 0.141
RT	1149.301; <0.001	1145.676; <0.001	2354.369; <0.001	55.316; <0.001	49.275; <0.001	117.764; <0.001	129.347; <0.001
TTFF	787.970; <0.001	12.581; 0.002	658.452; <0.001	41.998; <0.001	33.197; <0.001	756.946; <0.001	64.023; <0.001
Target fixation	267.106; <0.001	0.088; 0.957	269.746; <0.001	17.425; <0.001	8.663; 0.013	228.551; <0.001	31.297; <0.001
Clutter fixation	552.322; <0.001	1225.683; <0.001	942.577; <0.001	22.112; <0.001	21.998; <0.001	827.112; <0.001	29.401; <0.001

**Table 3:** GEE results for gaze-path measures. Each cell reports Wald chi-square; *p*.

Outcome	Salience	Layout	Clutter	S × L	S × C	L × C	S × L × C
C to T probability	2.659; 0.103	64.719; <0.001	8.140; 0.017	0.278; 0.870	0.180; 0.914	138.595; <0.001	1.965; 0.742
C to C probability	2.512; 0.113	92.251; <0.001	6.655; 0.036	2.471; 0.291	1.949; 0.377	197.320; <0.001	1.728; 0.786
Transitions before target	3.770; 0.052	87.558; <0.001	21.167; <0.001	8.617; 0.013	0.943; 0.624	212.272; <0.001	6.421; 0.170

Note: C-to-T and C-to-C probabilities were fitted with binomial GEE models, with responses clustered by participant. A separate Gaussian GEE was used for the number of transitions preceding the first Target-AOI visit after applying the transformation ln(1 + x).

## Data Availability

The data supporting the findings of this study are available from the corresponding author upon reasonable request.

## Supplementary Materials

The following supporting information can be downloaded at https://www.mdpi.com/article/10.3390/jemr19050106/s1, Figure S1: Stimulus matrix for the additional viewing angles; Table S1: Descriptive statistics for behavioral and eye-movement outcomes across experimental conditions; Table S2: Bonferroni-adjusted pairwise comparisons among clutter levels for continuous outcomes; Table S3: Valid-trial distribution, sensitivity analyses, and model-sample audit; Table S4: Condition-level gaze-transition metrics; Table S5: Complete gaze-transition matrices across spatial-organization and visual-clutter conditions. Supplementary Methods S6: Model specifications, preprocessing, sample-size planning, and measure inventory; Table S7: Interaction estimates and confidence intervals; Table S8: Random-slope sensitivity and diagnostics. Supplementary Methods S6.4: GEE specification; Table S9: Gaze-transition interaction contrasts.
