# Supplementary material for: Stage-Specific Effects of Spatial Organization on Visual Search Under Clutter: Behavioral and Eye-Movement Evidence
Source: J Eye Mov Res. 2026 Sep 21;19(5):106. doi: 10.3390/jemr19050106 (PMC13706149; doi:10.3390/jemr19050106)
Supplement: Supplementary file 1 [file jemr-19-00106-s001.zip › jemr-4567289-supplementary.pdf]

Supplementary Materials

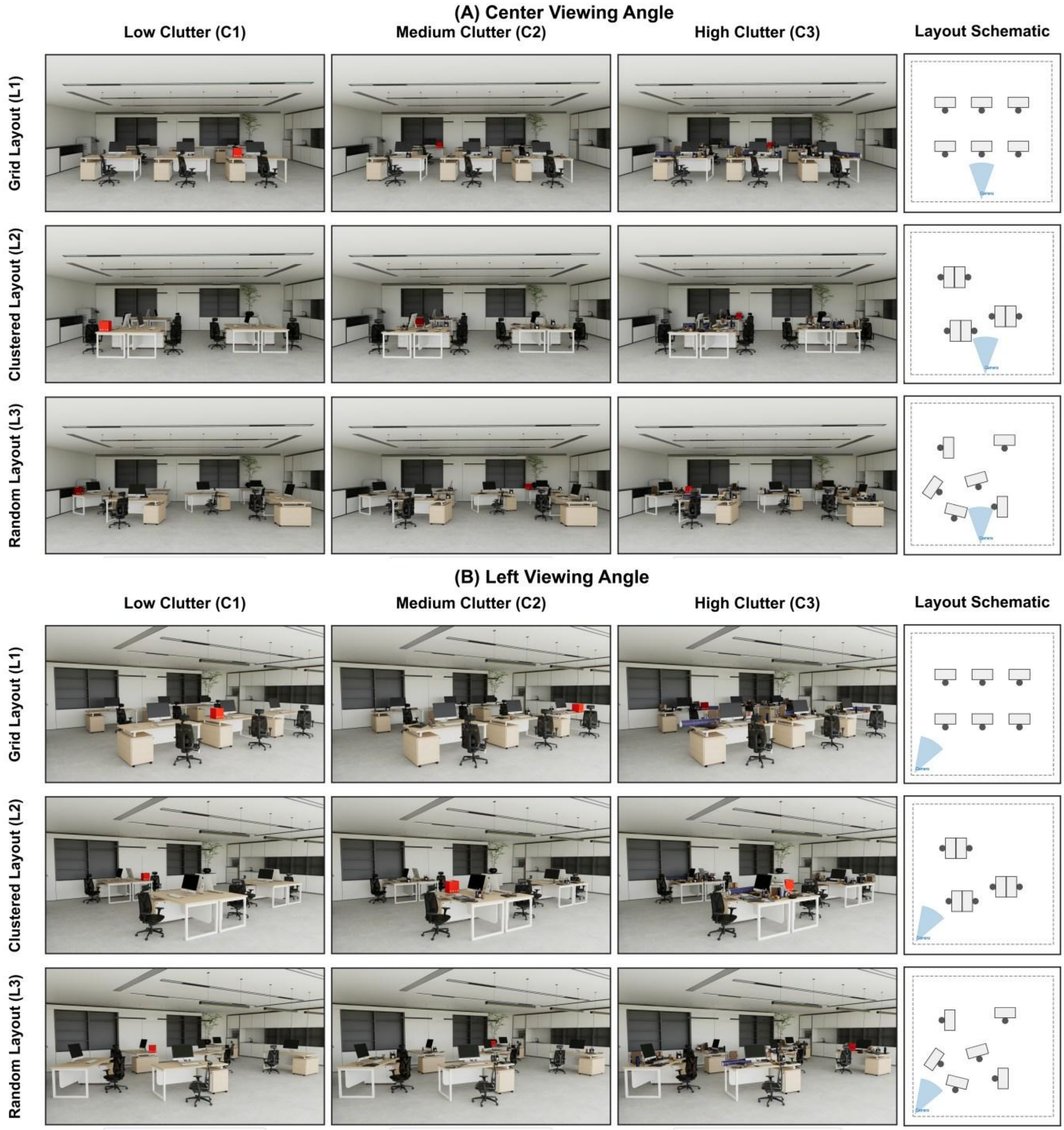

Figure S1. Stimulus matrix for the additional viewing angles. Representative stimuli under the center viewing angle (A) and left viewing angle (B), showing the three layout conditions (grid layout, L1; clustered layout, L2; random layout, L3) across the three clutter levels (low clutter, C1; medium clutter, C2; high clutter, C3). The rightmost column presents the corresponding layout schematics for each layout condition and viewing angle. In the high-salience condition shown here, the target object was a red upright folder. In the low-salience condition, the same object and scene configuration were used, with only the target color changed to black.

Table S1. Descriptive statistics for behavioral and eye-movement outcomes across experimental conditions.

Outcome	Target salience	Spatial organization	Low clutter (M ± SD)	Medium clutter (M ± SD)	High clutter (M ± SD)
Accuracy	High	Grid	84.7% ± 19.6%	86.1% ± 21.8%	80.6% ± 21.8%
Accuracy	High	Clustered	84.7% ± 17.0%	76.4% ± 20.8%	90.3% ± 18.3%
Accuracy	High	Random	77.8% ± 25.4%	84.7% ± 21.9%	77.8% ± 28.9%
Accuracy	Low	Grid	84.7% ± 17.0%	83.3% ± 17.0%	84.7% ± 19.6%
Accuracy	Low	Clustered	80.6% ± 23.9%	87.5% ± 16.5%	81.9% ± 21.9%
Accuracy	Low	Random	81.9% ± 24.0%	79.2% ± 23.7%	76.4% ± 23.0%
RT (ms)	High	Grid	1044.7 ± 96.8	1345.4 ± 142.9	1666.7 ± 112.6
RT (ms)	High	Clustered	1222.0 ± 116.4	1521.6 ± 123.6	1975.7 ± 139.8
RT (ms)	High	Random	1433.5 ± 155.7	1776.6 ± 179.0	2239.9 ± 201.6
RT (ms)	Low	Grid	1432.5 ± 147.1	1853.7 ± 185.8	2282.8 ± 275.9
RT (ms)	Low	Clustered	1731.5 ± 200.4	2166.9 ± 181.6	2753.8 ± 303.3
RT (ms)	Low	Random	2032.4 ± 177.7	2404.4 ± 194.9	4804.7 ± 388.8
TTFF (ms)	High	Grid	201.4 ± 33.0	236.3 ± 26.5	524.3 ± 56.1
TTFF (ms)	High	Clustered	241.8 ± 31.4	296.0 ± 34.5	356.1 ± 45.2
TTFF (ms)	High	Random	261.4 ± 40.1	319.4 ± 47.9	202.4 ± 46.7
TTFF (ms)	Low	Grid	287.6 ± 35.8	345.1 ± 57.5	724.5 ± 68.7
TTFF (ms)	Low	Clustered	317.3 ± 29.3	400.9 ± 56.7	505.4 ± 76.8
TTFF (ms)	Low	Random	365.0 ± 53.4	473.7 ± 63.2	457.1 ± 60.4
Clutter-AOI fixation duration (ms)	High	Grid	396.0 ± 98.7	494.9 ± 77.8	431.2 ± 96.1
Clutter-AOI fixation duration (ms)	High	Clustered	498.4 ± 95.4	573.6 ± 95.8	737.7 ± 91.4
Clutter-AOI fixation duration (ms)	High	Random	544.6 ± 96.6	644.6 ± 119.6	1682.3 ± 181.5
Clutter-AOI fixation duration (ms)	Low	Grid	539.8 ± 103.5	722.6 ± 136.4	631.2 ± 119.2
Clutter-AOI fixation duration (ms)	Low	Clustered	658.4 ± 123.0	846.2 ± 119.7	1074.9 ± 170.2
Clutter-AOI fixation duration (ms)	Low	Random	801.6 ± 100.0	931.7 ± 111.5	3842.5 ± 358.2
Target-AOI fixation duration (ms)	High	Grid	251.8 ± 78.9	268.3 ± 71.3	577.9 ± 84.6
Target-AOI fixation duration (ms)	High	Clustered	261.8 ± 57.4	312.9 ± 81.1	424.6 ± 60.0
Target-AOI fixation duration (ms)	High	Random	298.8 ± 78.3	356.4 ± 76.2	234.3 ± 77.5
Target-AOI fixation duration (ms)	Low	Grid	294.0 ± 77.5	394.3 ± 81.1	777.5 ± 156.2
Target-AOI fixation duration (ms)	Low	Clustered	349.3 ± 77.4	461.4 ± 80.2	593.6 ± 133.1
Target-AOI fixation duration (ms)	Low	Random	437.2 ± 70.7	528.6 ± 73.8	540.3 ± 61.7

Note: Accuracy values are participant-level condition proportions. Continuous outcomes are participant-level means calculated from correct valid trials and are reported in milliseconds. M = mean; SD = standard deviation.

Table S2. Bonferroni-adjusted pairwise comparisons among visual-clutter levels for continuous outcomes.

Outcome	Salience	Layout	Contrast	Ratio	95% CI	z	Bonferroni-adjusted p
RT (ms)	High	Grid	Low - Medium	0.773	[0.734, 0.814]	-9.838	<0.001
RT (ms)	High	Grid	Low - High	0.618	[0.587, 0.651]	-18.054	<0.001
RT (ms)	High	Grid	Medium - High	0.800	[0.759, 0.843]	-8.419	<0.001
RT (ms)	High	Clustered	Low - Medium	0.795	[0.754, 0.839]	-8.475	<0.001
RT (ms)	High	Clustered	Low - High	0.620	[0.590, 0.653]	-18.432	<0.001
RT (ms)	High	Clustered	Medium - High	0.780	[0.741, 0.822]	-9.316	<0.001
RT (ms)	High	Random	Low - Medium	0.824	[0.782, 0.869]	-7.202	<0.001
RT (ms)	High	Random	Low - High	0.647	[0.613, 0.683]	-15.825	<0.001
RT (ms)	High	Random	Medium - High	0.785	[0.745, 0.828]	-8.974	<0.001
RT (ms)	Low	Grid	Low - Medium	0.774	[0.735, 0.815]	-9.719	<0.001
RT (ms)	Low	Grid	Low - High	0.636	[0.604, 0.669]	-17.216	<0.001
RT (ms)	Low	Grid	Medium - High	0.822	[0.780, 0.866]	-7.425	<0.001
RT (ms)	Low	Clustered	Low - Medium	0.800	[0.760, 0.843]	-8.432	<0.001
RT (ms)	Low	Clustered	Low - High	0.632	[0.599, 0.666]	-17.077	<0.001
RT (ms)	Low	Clustered	Medium - High	0.790	[0.750, 0.831]	-8.978	<0.001
RT (ms)	Low	Random	Low - Medium	0.846	[0.803, 0.892]	-6.184	<0.001
RT (ms)	Low	Random	Low - High	0.422	[0.400, 0.446]	-31.614	<0.001
RT (ms)	Low	Random	Medium - High	0.499	[0.473, 0.527]	-25.290	<0.001
TTFF (ms)	High	Grid	Low - Medium	0.851	[0.784, 0.923]	-3.888	<0.001
TTFF (ms)	High	Grid	Low - High	0.380	[0.350, 0.413]	-22.872	<0.001
TTFF (ms)	High	Grid	Medium - High	0.447	[0.411, 0.485]	-19.130	<0.001
TTFF (ms)	High	Clustered	Low - Medium	0.800	[0.735, 0.870]	-5.204	<0.001
TTFF (ms)	High	Clustered	Low - High	0.669	[0.617, 0.725]	-9.784	<0.001
TTFF (ms)	High	Clustered	Medium - High	0.836	[0.770, 0.908]	-4.234	<0.001
TTFF (ms)	High	Random	Low - Medium	0.843	[0.775, 0.916]	-4.010	<0.001
TTFF (ms)	High	Random	Low - High	1.375	[1.262, 1.497]	7.307	<0.001
TTFF (ms)	High	Random	Medium - High	1.631	[1.501, 1.774]	11.469	<0.001
TTFF (ms)	Low	Grid	Low - Medium	0.851	[0.784, 0.924]	-3.848	<0.001
TTFF (ms)	Low	Grid	Low - High	0.397	[0.366, 0.431]	-22.097	<0.001
TTFF (ms)	Low	Grid	Medium - High	0.467	[0.430, 0.507]	-18.154	<0.001
TTFF (ms)	Low	Clustered	Low - Medium	0.799	[0.736, 0.868]	-5.345	<0.001
TTFF (ms)	Low	Clustered	Low - High	0.635	[0.584, 0.690]	-10.646	<0.001
TTFF (ms)	Low	Clustered	Medium - High	0.795	[0.732, 0.863]	-5.498	<0.001
TTFF (ms)	Low	Random	Low - Medium	0.763	[0.702, 0.830]	-6.311	<0.001
TTFF (ms)	Low	Random	Low - High	0.790	[0.725, 0.859]	-5.463	<0.001
TTFF (ms)	Low	Random	Medium - High	1.035	[0.950, 1.127]	0.780	1.000
Clutter-AOI fixation duration (ms)	High	Grid	Low - Medium	0.791	[0.714, 0.875]	-4.531	<0.001
Clutter-AOI fixation duration (ms)	High	Grid	Low - High	0.895	[0.807, 0.992]	-2.113	0.104
Clutter-AOI fixation duration (ms)	High	Grid	Medium - High	1.132	[1.021, 1.254]	2.352	0.056
Clutter-AOI fixation duration (ms)	High	Clustered	Low - Medium	0.843	[0.759, 0.936]	-3.191	0.004
Clutter-AOI fixation duration (ms)	High	Clustered	Low - High	0.664	[0.600, 0.734]	-7.988	<0.001
Clutter-AOI fixation duration (ms)	High	Clustered	Medium - High	0.788	[0.710, 0.873]	-4.529	<0.001
Clutter-AOI fixation duration (ms)	High	Random	Low - Medium	0.846	[0.762, 0.939]	-3.151	0.005
Clutter-AOI fixation duration (ms)	High	Random	Low - High	0.319	[0.287, 0.355]	-21.008	<0.001

Outcome	Salience	Layout	Contrast	Ratio	95% CI	z	Bonferroni-adjusted p
Clutter-AOI fixation duration (ms)	High	Random	Medium - High	0.378	[0.340, 0.419]	-18.295	<0.001
Clutter-AOI fixation duration (ms)	Low	Grid	Low - Medium	0.748	[0.676, 0.829]	-5.541	<0.001
Clutter-AOI fixation duration (ms)	Low	Grid	Low - High	0.867	[0.783, 0.960]	-2.750	0.018
Clutter-AOI fixation duration (ms)	Low	Grid	Medium - High	1.158	[1.045, 1.283]	2.803	0.015
Clutter-AOI fixation duration (ms)	Low	Clustered	Low - Medium	0.755	[0.681, 0.836]	-5.372	<0.001
Clutter-AOI fixation duration (ms)	Low	Clustered	Low - High	0.595	[0.536, 0.660]	-9.762	<0.001
Clutter-AOI fixation duration (ms)	Low	Clustered	Medium - High	0.788	[0.711, 0.873]	-4.573	<0.001
Clutter-AOI fixation duration (ms)	Low	Random	Low - Medium	0.861	[0.775, 0.956]	-2.811	0.015
Clutter-AOI fixation duration (ms)	Low	Random	Low - High	0.205	[0.184, 0.228]	-29.383	<0.001
Clutter-AOI fixation duration (ms)	Low	Random	Medium - High	0.238	[0.214, 0.265]	-26.378	<0.001
Target-AOI fixation duration (ms)	High	Grid	Low - Medium	0.838	[0.723, 0.970]	-2.362	0.055
Target-AOI fixation duration (ms)	High	Grid	Low - High	0.378	[0.326, 0.439]	-12.778	<0.001
Target-AOI fixation duration (ms)	High	Grid	Medium - High	0.452	[0.389, 0.524]	-10.491	<0.001
Target-AOI fixation duration (ms)	High	Clustered	Low - Medium	0.768	[0.660, 0.894]	-3.405	0.002
Target-AOI fixation duration (ms)	High	Clustered	Low - High	0.542	[0.469, 0.627]	-8.251	<0.001
Target-AOI fixation duration (ms)	High	Clustered	Medium - High	0.705	[0.608, 0.818]	-4.612	<0.001
Target-AOI fixation duration (ms)	High	Random	Low - Medium	0.802	[0.690, 0.932]	-2.885	0.012
Target-AOI fixation duration (ms)	High	Random	Low - High	1.374	[1.180, 1.602]	4.076	<0.001
Target-AOI fixation duration (ms)	High	Random	Medium - High	1.714	[1.475, 1.991]	7.039	<0.001
Target-AOI fixation duration (ms)	Low	Grid	Low - Medium	0.760	[0.656, 0.880]	-3.656	<0.001
Target-AOI fixation duration (ms)	Low	Grid	Low - High	0.378	[0.326, 0.437]	-13.029	<0.001
Target-AOI fixation duration (ms)	Low	Grid	Medium - High	0.497	[0.429, 0.576]	-9.316	<0.001
Target-AOI fixation duration (ms)	Low	Clustered	Low - Medium	0.731	[0.631, 0.847]	-4.171	<0.001
Target-AOI fixation duration (ms)	Low	Clustered	Low - High	0.570	[0.490, 0.661]	-7.374	<0.001
Target-AOI fixation duration (ms)	Low	Clustered	Medium - High	0.779	[0.673, 0.902]	-3.336	0.003
Target-AOI fixation duration (ms)	Low	Random	Low - Medium	0.805	[0.693, 0.935]	-2.831	0.014
Target-AOI fixation duration (ms)	Low	Random	Low - High	0.771	[0.662, 0.897]	-3.358	0.002
Target-AOI fixation duration (ms)	Low	Random	Medium - High	0.958	[0.822, 1.116]	-0.549	1.000

Note: Continuous outcomes were analyzed on the natural-log scale. Ratios and pointwise 95% CIs are back-transformed from log-scale contrasts. Bonferroni adjustment applies to p values across the three clutter comparisons within each salience × layout cell; the CIs are not multiplicity-adjusted.

Table S3. Valid-trial distribution, sensitivity analyses, and model-sample audit.

A. Distribution of correct-trial counts per participant × spatial-organization × visual-clutter cell

Target salience	0 trials	1 trial	2 trials	3 trials	Total cells	Cells with ≥2 trials (%)
High	1	17	76	122	216	91.7
Low	1	12	88	115	216	94.0
Overall	2	29	164	237	432	92.8

B. Robustness of the key layout × clutter and three-way effects

Outcome	Effect	Original χ^2	p	≥2-trial cell-mean χ^2	p	Complete 3-trial cell χ^2	p
RT	Layout × clutter	117.764	<0.001	111.994	<0.001	71.792	<0.001
RT	Salience × layout × clutter	129.347	<0.001	115.420	<0.001	86.337	<0.001
TTFF	Layout × clutter	756.946	<0.001	748.615	<0.001	489.117	<0.001
TTFF	Salience × layout × clutter	64.023	<0.001	69.130	<0.001	43.847	<0.001
Clutter-AOI fixation duration	Layout × clutter	827.112	<0.001	743.569	<0.001	473.403	<0.001
Clutter-AOI fixation duration	Salience × layout × clutter	29.401	<0.001	25.307	<0.001	20.413	<0.001
Target-AOI fixation duration	Layout × clutter	228.551	<0.001	236.773	<0.001	141.093	<0.001
Target-AOI fixation duration	Salience × layout × clutter	31.297	<0.001	27.858	<0.001	27.253	<0.001

C. Model-sample audit

Outcome	Analysis set	Trials/cells n	Participants n	Retained cells	Excluded sparse cells
RT	Original trial-level	1068	48		
RT	Trial-level, cells ≥2	1039	48	401	29
RT	Cell means, cells ≥2	401	48	401	29
RT	Complete 3-trial cell means	237	48	237	193
TTFF	Original trial-level	1068	48		
TTFF	Trial-level, cells ≥2	1039	48	401	29
TTFF	Cell means, cells ≥2	401	48	401	29
TTFF	Complete 3-trial cell means	237	48	237	193
Clutter-AOI fixation duration	Original trial-level	1068	48		
Clutter-AOI fixation duration	Trial-level, cells ≥2	1039	48	401	29
Clutter-AOI fixation duration	Cell means, cells ≥2	401	48	401	29
Clutter-AOI fixation duration	Complete 3-trial cell means	237	48	237	193
Target-AOI fixation duration	Original trial-level	1064	48		
Target-AOI fixation duration	Trial-level, cells ≥2	1033	48	399	31
Target-AOI fixation duration	Cell means, cells ≥2	399	48	399	31
Target-AOI fixation duration	Complete 3-trial cell means	235	48	235	195

Note: The main sensitivity analysis retained cells with at least two valid correct trials, averaged the log-transformed outcome within each cell, and refitted the random-intercept model. The stricter analysis required all three viewing-angle trials to be valid. Excluded sparse cells are counted among cells with at least one usable observation; zero-observation cells are shown in panel A.

Table S3. Continued

D. Acquisition and analytic exclusions

Stage	Count	Explanation
Planned target-present trials	1,296	48 participants × 27 trials
Additional presentations	12	One successful repeat for each quality-failed presentation
All target-present presentations	1,308	Includes repeats; excludes target-absent trials
Original quality-failed records removed	12	Below 80% valid gaze samples; replaced by successful repeats
Retained accuracy observations	1,296	One record per planned target-present trial
Incorrect responses excluded from continuous models	228	Incorrect target selections
RT, TTFF, Clutter duration	1,068	Correct trials with positive finite values
Target duration exclusions	4	Three negative values and one zero
Target duration model	1,064	48 participants retained

Table S4. Condition-level gaze-transition metrics across target-salience, spatial-organization, and visual-clutter conditions.

Salience	Layout	Clutter	Clutter outgoing n	C→T (%)	C→C (%)	Participants n	Transitions before target (M ± SD)
High	Grid	Low	112	28.6	32.1	24	1.78 ± 0.89
High	Grid	Medium	162	29.6	37.0	24	1.98 ± 1.12
High	Grid	High	116	50.0	18.1	24	1.01 ± 0.73
High	Clustered	Low	127	33.1	31.5	24	1.67 ± 0.95
High	Clustered	Medium	177	31.1	35.0	24	2.51 ± 1.50
High	Clustered	High	266	29.7	36.1	24	1.91 ± 1.35
High	Random	Low	145	31.0	36.6	24	1.65 ± 1.20
High	Random	Medium	222	30.6	37.8	24	1.87 ± 1.24
High	Random	High	408	10.5	64.5	23	5.09 ± 2.42
Low	Grid	Low	177	29.4	39.0	24	1.53 ± 1.03
Low	Grid	Medium	231	25.1	36.8	24	2.27 ± 1.24
Low	Grid	High	201	45.8	21.9	24	0.78 ± 0.81
Low	Clustered	Low	192	29.2	34.9	24	1.72 ± 1.33
Low	Clustered	Medium	261	29.5	37.9	24	2.27 ± 1.24
Low	Clustered	High	323	26.9	36.8	24	2.49 ± 1.65
Low	Random	Low	225	27.6	38.7	24	2.10 ± 0.95
Low	Random	Medium	276	28.6	33.7	24	2.28 ± 1.58
Low	Random	High	954	11.4	64.0	23	7.69 ± 4.07

Note: C→T and C→C probabilities are pooled observed transitions leaving Clutter within each condition. Transitions before target are participant-condition means; participants without an estimable target-reach value were excluded from that condition mean. M = mean; SD = standard deviation.

Table S5. Complete gaze-transition matrices across spatial-organization and visual-clutter conditions, pooled across target-salience groups.

Clutter	Layout	Origin	Target (%)	Clutter (%)	Background (%)	Outgoing n
Low	Grid	Target	12.4	47.6	40.0	185
Low	Grid	Clutter	29.1	36.3	34.6	289
Low	Grid	Background	35.5	45.3	19.2	214
Low	Clustered	Target	16.9	45.8	37.3	201
Low	Clustered	Clutter	30.7	33.5	35.7	319
Low	Clustered	Background	27.8	50.2	22.0	241
Low	Random	Target	21.2	45.0	33.8	240
Low	Random	Clutter	28.9	37.8	33.2	370
Low	Random	Background	29.2	47.8	23.0	274
Medium	Grid	Target	18.2	48.7	33.1	236
Medium	Grid	Clutter	27.0	36.9	36.1	393
Medium	Grid	Background	31.4	45.7	22.9	280
Medium	Clustered	Target	25.1	41.0	33.9	283
Medium	Clustered	Clutter	30.1	36.8	33.1	438
Medium	Clustered	Background	27.2	44.9	27.9	323
Medium	Random	Target	18.0	45.7	36.3	317
Medium	Random	Clutter	29.5	35.5	34.9	498
Medium	Random	Background	29.7	48.3	22.0	381
High	Grid	Target	39.2	27.8	33.0	543
High	Grid	Clutter	47.3	20.5	32.2	317
High	Grid	Background	46.2	30.5	23.4	364
High	Clustered	Target	20.9	43.7	35.4	378
High	Clustered	Clutter	28.2	36.5	35.3	589
High	Clustered	Background	28.7	44.3	27.0	470
High	Random	Target	7.2	62.9	29.9	221
High	Random	Clutter	11.2	64.2	24.7	1362
High	Random	Background	10.2	67.4	22.4	522

Note: Matrices are based on observed adjacent fixation-state transitions and pooled across salience groups. Rows sum to 100% apart from rounding. T = Target; C = Clutter; B = Background.

Supplementary Methods S6. Processing and model specifications

S6.1. Gaze processing and measure inventory

The I-VT Fixation settings were: 30 degrees/s velocity threshold; 20-ms velocity window; three-sample moving median; Average eye selection; interpolation disabled; adjacent-fixation merging enabled (maximum 75 ms and 0.5 degrees); minimum fixation duration 60 ms. Invalid and off-screen coordinates were not coded as Background. Target and Clutter followed object outlines and were mutually exclusive; remaining valid image positions were Background. Nine-point calibration was followed by validation with mean error ≤0.5 degrees and inspection across locations. Trials required ≥80% valid samples. Twelve unsuccessful presentations were replaced by successful repeats, as accounted for in Table S3D.

The Tobii Pro Lab User Manual v1.162, Appendix B, describes the I-VT configuration and processing functions (https://s3.amazonaws.com/lynx.tobii/TobiiProLab_1_162_UserManual.pdf). The study used version 24.21.435.0.

Measure group	Collected variables	Use in this report
Pre-task questionnaire	Age, occupation, vision and color-vision difficulties	Sample description; not model predictors
Focal behavioral and eye measures	Accuracy, RT, target TTFF, Target and Clutter total fixation duration	Primary and secondary models
Additional eye metrics	Overall TTFF, duration, fixation and saccade counts; Target fixation/saccade counts; Clutter TTFF and fixation/saccade counts; Background TTFF, duration, fixation/saccade counts	Collected and included in preliminary processing; not reported as focal analyses
Gaze-state measures	C→T, C→C and transitions before target	Search-path analyses
NASA-TLX	Six dimensions, rated 1-20 for each image after completion of all search trials	Collected; excluded from this report; no workload total or inference added

S6.2. Sample-size planning

An a priori sample-size calculation was conducted in G*Power using the repeated-measures ANOVA within-between interaction option. The planning assumptions were f = 0.15, alpha = 0.05, power = 0.80, two groups, nine repeated conditions, a repeated-measures correlation of 0.50, and a nonsphericity correction of 0.75. The minimum total sample was 46; the final sample comprised 48 participants, 24 per group. This calculation approximated the overall interaction between salience group and the nine layout-by-clutter conditions, not the power of each specific interaction in the final mixed-effects models (Supplementary Methods S6).

Reference: Faul F, Erdfelder E, Lang AG, Buchner A. G*Power 3: A flexible statistical power analysis program for the social, behavioral, and biomedical sciences. Behavior Research Methods. 2007;39:175-191. https://doi.org/10.3758/BF03193146. The planning effect size was f = 0.15. Numerical recalculation gives power approximately 0.801 at N = 46 and 0.821 at N = 48 under these assumptions.

Supplementary Methods S6. Continued

S6.3. Mixed-effects specifications and inference

Let S denote salience, L layout, C clutter, V viewing angle, i participant, and j trial. The fixed-effects design is defined by Equation (S1), using sum-to-zero contrast columns and their interaction products. The continuous and accuracy models are given in Equations (S2) and (S3).

$$x_{ij} = (1, S, L, C, V, S \otimes L, S \otimes C, L \otimes C, S \otimes L \otimes C)_{ij} \quad \text{(S1)}$$

$$ln(Y_{ij}) = x_{ij}{}^{T}\beta + b_i + \varepsilon_{ij} \quad \text{(S2)}$$

$$logit[P(A_{ij} = 1 \mid b_i)] = x_{ij}{}^{T}\beta + b_i \quad \text{(S3)}$$

The symbol ⊗ denotes all pairwise products of the corresponding contrast columns. The random intercept b_i is Gaussian with mean zero and variance τ^2; ε_{ij} is an independent Gaussian residual with mean zero and variance σ^2.

Factor	Level order	Coding
Salience	High, Low	+1, -1
Layout	Grid, Clustered, Random	(1,0), (0,1), (-1,-1)
Clutter	Low, Medium, High	(1,0), (0,1), (-1,-1)
View	1, 2, 3	(1,0), (0,1), (-1,-1)

There is no treatment-reference level: effects use sum coding. Interaction columns are products of the corresponding contrasts. Continuous models use REML. Accuracy uses maximum likelihood with 25-point Gauss-Hermite quadrature; its random-intercept variance reached zero, where the fit agrees with ordinary logistic regression. Models were fitted using Python 3.12.14, NumPy 2.3.5, SciPy 1.17.0, and pandas 2.2.3.

For a term-selection matrix A, the Wald statistic in Equation (S4) is compared with χ^2 with rank(A) degrees of freedom. $\hat{V}\beta$ is the fixed-effect block of the inverse observed joint Hessian. Equation (S5) gives the standard error of a single contrast v; Equation (S6) gives its pointwise 95% ratio interval. Marginal means average equally over the three views on the log scale and are geometric means after exponentiation.

$$W = (A\hat{\beta})^{T}[A\,\hat{V}_{\beta}\,A^{T}]^{-1}(A\hat{\beta}) \quad \text{(S4)}$$

$$SE(v^{T}\hat{\beta}) = \sqrt{(v^{T}\,\hat{V}_{\beta}\,v)} \quad \text{(S5)}$$

$$CI_{95}\% = exp[v^{T}\hat{\beta} \pm 1.959964 \times SE(v^{T}\hat{\beta})] \quad \text{(S6)}$$

Random-slope models use z = (1, L1, L2, C1, C2) for each participant. DIAG estimates five independent variances; FULL estimates the full correlated covariance. The fixed effects and outcome-specific samples are unchanged. For comparison across random structures, Table S8 uses plug-in GLS covariance conditional on the fitted random covariance; this differs from the observed-Hessian convention for the primary tests. The binomial slope model uses independent coefficients and a Laplace approximation. Convergence and covariance boundaries are separate diagnostics.

Table S7. Interaction estimates and pointwise 95% confidence intervals

R = (high-clutter/low-clutter geometric mean in the first layout)/(the same ratio in the second layout), within salience. Q = R_low-salience/R_high-salience. R quantifies a layout × clutter contrast; Q quantifies its change with salience. The null value is 1. Six R contrasts per outcome form one Bonferroni family; three Q contrasts form another. Confidence intervals are pointwise, while p values are adjusted. These contrasts quantify specific interactions between condition comparisons; they are not effect sizes for the entire omnibus interaction.

RT

Contrast	Salience	Layout pair	Ratio	95% CI	Adjusted p
R	High	Grid/Clustered	1.004	[0.933, 1.080]	1.000
R	Low	Grid/Clustered	0.994	[0.923, 1.070]	1.000
Q	Low/High	Grid/Clustered	0.990	[0.892, 1.098]	1.000
R	High	Grid/Random	1.047	[0.971, 1.129]	1.000
R	Low	Grid/Random	0.664	[0.617, 0.715]	<0.001
Q	Low/High	Grid/Random	0.634	[0.571, 0.705]	<0.001
R	High	Clustered/Random	1.043	[0.968, 1.123]	1.000
R	Low	Clustered/Random	0.669	[0.620, 0.721]	<0.001
Q	Low/High	Clustered/Random	0.641	[0.577, 0.712]	<0.001

TTFF

Contrast	Salience	Layout pair	Ratio	95% CI	Adjusted p
R	High	Grid/Clustered	1.760	[1.568, 1.976]	<0.001
R	Low	Grid/Clustered	1.598	[1.421, 1.796]	<0.001
Q	Low/High	Grid/Clustered	0.908	[0.770, 1.070]	0.745
R	High	Grid/Random	3.618	[3.212, 4.075]	<0.001
R	Low	Grid/Random	1.986	[1.766, 2.235]	<0.001
Q	Low/High	Grid/Random	0.549	[0.464, 0.649]	<0.001
R	High	Clustered/Random	2.055	[1.828, 2.311]	<0.001
R	Low	Clustered/Random	1.243	[1.104, 1.401]	0.002
Q	Low/High	Clustered/Random	0.605	[0.512, 0.715]	<0.001

Table S7. Continued

Clutter TFD

Contrast	Salience	Layout pair	Ratio	95% CI	Adjusted p
R	High	Grid/Clustered	0.742	[0.643, 0.857]	<0.001
R	Low	Grid/Clustered	0.686	[0.593, 0.794]	<0.001
Q	Low/High	Grid/Clustered	0.925	[0.753, 1.135]	1.000
R	High	Grid/Random	0.357	[0.308, 0.414]	<0.001
R	Low	Grid/Random	0.237	[0.204, 0.274]	<0.001
Q	Low/High	Grid/Random	0.663	[0.538, 0.817]	<0.001
R	High	Clustered/Random	0.481	[0.415, 0.557]	<0.001
R	Low	Clustered/Random	0.345	[0.297, 0.400]	<0.001
Q	Low/High	Clustered/Random	0.717	[0.582, 0.883]	0.005

Target TFD

Contrast	Salience	Layout pair	Ratio	95% CI	Adjusted p
R	High	Grid/Clustered	1.432	[1.163, 1.764]	0.004
R	Low	Grid/Clustered	1.508	[1.223, 1.860]	<0.001
Q	Low/High	Grid/Clustered	1.053	[0.784, 1.415]	1.000
R	High	Grid/Random	3.632	[2.934, 4.497]	<0.001
R	Low	Grid/Random	2.042	[1.654, 2.522]	<0.001
Q	Low/High	Grid/Random	0.562	[0.416, 0.759]	<0.001
R	High	Clustered/Random	2.536	[2.053, 3.132]	<0.001
R	Low	Clustered/Random	1.354	[1.094, 1.675]	0.032
Q	Low/High	Clustered/Random	0.534	[0.395, 0.721]	<0.001

Table S8. Random-slope sensitivity and model diagnostics

Outcome	Structure	L × C χ^2	S × L × C χ^2	Both p	Converged	Boundary
RT	RI	117.771	129.353	<0.001	Yes	No
RT	DIAG	117.778	129.446	<0.001	Yes	Yes
RT	FULL	119.317	131.741	<0.001	Yes	Yes
TTFF	RI	757.566	64.024	<0.001	Yes	Yes
TTFF	DIAG	760.662	64.096	<0.001	Yes	Yes
TTFF	FULL	775.452	64.104	<0.001	Yes	Yes
Target TFD	RI	228.555	31.302	<0.001	Yes	Yes
Target TFD	DIAG	229.030	31.320	<0.001	Yes	Yes
Target TFD	FULL	233.949	31.802	<0.001	Yes	Yes
Clutter TFD	RI	827.113	29.401	<0.001	Yes	Yes
Clutter TFD	DIAG	831.910	29.641	<0.001	Yes	Yes
Clutter TFD	FULL	866.358	30.585	<0.001	Yes	Yes

RI: random intercept; DIAG: independent intercept and four categorical slopes; FULL: correlated intercept and slopes. All listed tests have 4 df. A boundary is flagged when the smallest eigenvalue of the random covariance relative to residual variance is below 10^{-6}. All slope fits converged, but DIAG models contained zero variance components and FULL models were rank deficient. This limits interpretation of individual variance estimates, although the focal fixed-effect conclusions were stable.

The independent-slope accuracy model retained a nonsignificant three-way interaction, $\chi^2(4) = 6.957$, $p = 0.138$. Two nonzero slope variances were approximately 0.05127 and 0.01978; the remaining variance components reached zero. The primary accuracy random-intercept variance was also zero. The sensitivity analysis does not establish that participant-specific slopes are absent.

Supplementary Methods S6.4. GEE specification and interaction contrasts

The supplementary GEE analyses and interaction contrasts were computed using Python 3.12.14, with NumPy 2.3.5, SciPy 1.17.0, and pandas 2.2.3. The accompanying code specifies the estimating equations and covariance calculation.

Both transition-probability models used a Bernoulli distribution and logit link, with each outgoing Clutter transition as an observation: the response was 1 for the specified destination (Target or Clutter) and 0 otherwise. Participant-condition transition counts supplied these binary frequencies. They represented 4,575 outgoing transitions in 429 participant-condition cells from 48 participants; three cells without an outgoing Clutter transition contributed no observations. This representation uses counts and does not require an ordering of transitions within a participant for the exchangeable correlation structure.

For the target-reach outcome, the response was $\ln(1 + k)$, where k was the participant-condition mean number of transitions preceding the first Target visit. A Gaussian GEE with an identity link used 430 participant-condition observations from 48 participants; two cells without an estimable target-reach value were omitted. No weighting by the number of correct trials was applied to this model.

All three mean models included salience, layout, clutter, and all two- and three-way interactions, with the sum contrasts in S6.3 and no viewing-angle term. Clustering was by participant. An exchangeable working correlation allowed a common within-participant correlation; the estimated correlations were −0.002605 for C-to-T, −0.003937 for C-to-C, and −0.052165 for the transformed target-reach outcome. Estimation alternated Fisher-scoring updates of the mean coefficients with Pearson-residual moment updates of the correlation. All three fits converged.

Inference used the empirical sandwich covariance: the inverse sensitivity matrix multiplied the sum of participant-level score outer products and the inverse sensitivity matrix on its two sides. No finite-cluster multiplier was applied. Wald tests used the degrees of freedom of the tested coefficient block (1 for salience, 2 for layout, clutter, and salience-by-layout or salience-by-clutter, and 4 for layout-by-clutter and the three-way interaction). The covariance retains correlations between conditions from the same participant.

For each layout pair, R compares the high-to-low clutter effect between the first and second layout, averaging the two salience-specific log contrasts with equal weights. Q compares that interaction contrast under low versus high salience. For the two probability outcomes, exponentiation gives a ratio of odds ratios, not a ratio of probabilities. For the target-reach outcome, it gives a ratio of ratios of geometric means of $(1 + k)$, not a ratio of untransformed counts. The null value is 1. Contrast standard errors use the same participant-clustered sandwich covariance. Pointwise 95% intervals are obtained by exponentiating the log contrast ±1.959964 times its standard error. Bonferroni adjustment applies separately to the three R tests and three Q tests per outcome. These decompositions are exploratory.

Table S9. Gaze-transition interaction contrasts and pointwise 95% confidence intervals

R: layout × clutter contrast averaged equally across salience groups on the model scale. Q: low-salience/high-salience ratio of the layout × clutter contrasts. Grid/Clustered, Grid/Random, and Clustered/Random identify the first and second layouts. Ratios for C-to-T and C-to-C are ratios of odds ratios; those for target reach concern the geometric mean of (1 + k).

Outcome	Contrast	Layout pair	Ratio	95% CI	Adjusted p
C-to-T probability	R	Grid/Clustered	2.597	[1.730, 3.899]	<0.001
C-to-T probability	R	Grid/Random	7.665	[4.929, 11.922]	<0.001
C-to-T probability	R	Clustered/Random	2.952	[2.166, 4.023]	<0.001
C-to-T probability	Q	Grid/Clustered	0.790	[0.350, 1.780]	1.000
C-to-T probability	Q	Grid/Random	0.636	[0.263, 1.539]	0.947
C-to-T probability	Q	Clustered/Random	0.805	[0.434, 1.496]	1.000
C-to-C probability	R	Grid/Clustered	0.396	[0.253, 0.621]	<0.001
C-to-C probability	R	Grid/Random	0.150	[0.097, 0.233]	<0.001
C-to-C probability	R	Clustered/Random	0.379	[0.277, 0.518]	<0.001
C-to-C probability	Q	Grid/Clustered	1.059	[0.431, 2.605]	1.000
C-to-C probability	Q	Grid/Random	1.005	[0.416, 2.430]	1.000
C-to-C probability	Q	Clustered/Random	0.949	[0.508, 1.772]	1.000
Transitions before target	R	Grid/Clustered	0.600	[0.454, 0.792]	<0.001
Transitions before target	R	Grid/Random	0.286	[0.226, 0.362]	<0.001
Transitions before target	R	Clustered/Random	0.477	[0.343, 0.663]	<0.001
Transitions before target	Q	Grid/Clustered	0.759	[0.435, 1.324]	0.995
Transitions before target	Q	Grid/Random	0.876	[0.545, 1.407]	1.000
Transitions before target	Q	Clustered/Random	1.154	[0.597, 2.230]	1.000

Note: Confidence intervals are pointwise; p values are Bonferroni-adjusted within the contrast families defined in S6.4. The R and Q estimates are specific components of the omnibus interactions. N = 48 participant clusters in all three models.
